# SputOMICs identifies common and distinct markers in cystic fibrosis and chronic obstructive pulmonary disease

**DOI:** 10.21203/rs.3.rs-6095597/v1

**Published:** 2025-08-20

**Authors:** Sebastien Boutin, Dario Frey, Barbara Helm, Matteo Guerra, Matthias Hagner, Junyan Lu, Susanne Dittrich, Sabine Wege, Ralf Eberhardt, Felix Herth, Olaf Sommerburg, Carsten Schultz, Alexander Dalpke, Ursula Klingmueller, Marcus Mall

**Affiliations:** Institute of Medical Microbiology, University of Lübeck and University Hospital Schleswig-Holstein, Campus Lübeck; German Cancer Research Center (DKFZ); German Cancer Research Center (DKFZ); Department of Translational Pulmonology, University of Heidelberg; Department of Translational Pulmonology, University of Heidelberg; Heidelberg; ThoraxKlinik, University Heidelberg; ThoraxKlinik, University Heidelberg; ThoraxKlinik, University Heidelberg; Thoraxklinik, University Hospital Heidelberg; Division of Pediatric Pulmonology & Allergology and Cystic Fibrosis Center, Department of Pediatrics, University of Heidelberg; Oregon Health and Science University; University Hospital Heidelberg; German Cancer Research Center (DKFZ); Charité - Universitätsmedizin Berlin

## Abstract

Cystic fibrosis (CF) and chronic obstructive pulmonary disease (COPD) are muco-obstructive lung diseases. Knowledge of molecular processes has much improved therapeutic options in CF, whereas much less is known for COPD, a disease affecting an increasing number of patients. Here, we report a multilayer workflow integrating microbiome, inflammation and proteome profiling with clinical data to identify disease specific characteristics in sputum. Our proof-of-concept study shows that CF sputum is dominated by *Pseudomonas* and *Staphylococcus*, exhibits heightened neutrophilic inflammation, and a severe protease-antiprotease imbalance. In contrast, COPD displays heterogeneous microbiome composition, eosinophilic inflammation, and altered extracellular matrix remodeling. Proteome-based cellular deconvolution identifies disease-specific immune cell signatures, underscoring the complexity, especially in COPD. Multi-omics factor analysis establishes matrisome, and nucleotide metabolism changes as key disease discriminators. These findings highlight the potential of our integrated approach to uncover sputum biomarkers as tools for patient stratification and personalized therapeutic strategies in CF and COPD.

## Introduction

Cystic fibrosis (CF) and chronic obstructive pulmonary disease (COPD) are chronic muco-obstructive lung diseases characterized by chronic neutrophilic airway inflammation and dysbiosis, leading to a protease-antiprotease imbalance and progressive structural lung damage^1, 2, 3^. Despite these shared pathological features, CF and COPD differ significantly in their underlying causes, clinical presentations, and therapeutic approaches.

CF is an autosomal recessive disorder caused by mutations in the cystic fibrosis transmembrane conductance regulator (CFTR) gene, which results in dysfunctional CFTR channels^2^. This defect causes abnormal mucus properties and impaired mucociliary clearance, fostering chronic airway infection, inflammation, and structural damage, collectively contributing to a gradual decline in lung function^1, 2^. In contrast, COPD is primarily an acquired disease caused by long-term exposure to harmful particles and gases, particularly tobacco smoke, but also occupational dust and air pollution^4^. Affecting over 391 million people globally, COPD is projected to become the leading cause of death worldwide within the next 15 years^4^

Therapeutic advancements for CF have been transformative in recent years, particularly with the development of CFTR modulators such as Elexacaftor/Tezacaftor/Ivacaftor^5, 6, 7, 8^. These therapies target the underlying molecular defect, resulting in substantially improved clinical outcomes for CF patients^5, 6^. In addition to improving pulmonary health, CFTR modulators have been shown to partially normalize the sputum proteome and increase microbiome diversity by reducing pathogenic dominance of classical pathogen such as *Pseudomonas aeruginosa*^9, 10^. On the contrary, progress in targeted therapies for COPD has lagged^3^. The diagnosis of COPD remains to be based on lung function measurements using forced spirometry to determine the forced expiratory volume in 1 second (FEV_1_)^11^. As specified by the Global Initiative for Chronic Obstructive Lung Disease (GOLD), COPD patients are subcategorized based on the FEV_1_ into stages ranging from mild (GOLD I) to very severe (GOLD IV)^12^. Moreover, COPD is frequently associated with severe comorbidities, including cardiovascular disease and metabolic syndrome, which complicate disease management and treatment strategies^13^. Although chronic inflammation and airway microbiota alterations in COPD are increasingly recognized as critical contributors to disease progression, research has predominantly focused on plasma and serum biomarkers^14, 15, 16, 17^. Sputum, a readily accessible and non-invasive sample, remains underutilized in advanced COPD research despite its potential to provide direct insights into airway-specific molecular and microbial changes.

Sputum analysis offers a unique and accessible view of the lower respiratory tract. It thus provides insights into the distinct pathophysiological mechanisms underlying chronic respiratory diseases such as CF and COPD. Unlike invasive methods, such as tissue biopsies or blood analyses, sputum collection is a non-invasive, patient-friendly approach that enables direct assessment of airway inflammation, immune cell profiles, protease activity, and microbiome dynamics. Lately, in CF, proteomic studies of sputum samples allowed us to identify proteomic changes during modulator therapy and to compare those to healthy individuals^9, 18^. Meanwhile, in COPD, most research has focused on analyzing plasma or serum samples, comparing stable patients with exacerbated patients^14, 15^. Up to now, only in a few studies, sputum samples from COPD patients have been examined, and in those, the emphasis was on comparing smokers with nonsmokers^16, 17^. While sputum analysis is particularly advantageous for evaluating disease states and therapeutic outcomes over time, current studies have focused mainly on individual parameters and have not yet integrated several layers of analysis nor performed a comparative analysis of both chronic muco-obstructive lung diseases.

CF and COPD involve chronic neutrophilic inflammation, with granule protein release contributing to persistent lung damage^19, 20^. However, the inflammatory profiles and clinical implications of these conditions are distinct. In CF, chronic infection and inflammation are closely linked, with therapeutic efforts targeting both aspects^8, 9^. In COPD, inflammation, exacerbations, and airway microbiota alterations are interconnected. Still, current treatments often fail to address these links adequately^13, 21, 22^. This unmet need for anti-inflammatory and anti-infective therapies, particularly those tailored to specific disease characteristics, underscores the importance of comprehensive, integrative approaches to disease characterization.

To address these gaps, we established an integrative SputOMICs workflow that was employed for a detailed comparative analysis of microbiome, inflammation, protease-antiprotease imbalance, and proteome in sputum samples from patients with CF or COPD, as well as from healthy controls in an observational study. Our multi-omics approach combines microbiome studies with mass spectrometry-based proteomics for direct quantification of protein abundance, revealing disease-specific changes in CF and COPD sputum, particularly proteins associated with adaptive immunity pathways and shifts in protease-antiprotease balance, suggesting a key role for microbiome alterations in both conditions but also provides evidence for distinct mechanisms. Integrating proteomic and microbiome insights, our study unravels the mechanistic complexities of CF and COPD. It identifies sputum markers that provide a basis to refine disease classification and pave the way for individualized treatment options.

## Material and methods

Additional information is provided in the online supplement.

### Study population

This prospective observational study was approved by the University of Heidelberg’s ethics committee (S-046/2009, S-370/2011, S-041/2018). All patients gave written informed consent. Table 1 provides demographics and clinical characteristics.

### Microbiome analysis

Samples used for the microbiome analysis were incubated with PMA^™^ dye (Biotium Inc., Hayward, USA). Subsequently, the copy number of the 16S rDNA gene was quantified, and libraries were prepared as published earlier^23^. The sequence data was processed using Dada2, and amplicon sequence variants (ASVs) were counted and classified using Silva database v138.1. The raw data are available in the SRA repository (PRJNA1078153).

### Sputum collection and sample pre-treatment for inflammatory biomarker analysis

**Figure 1:**
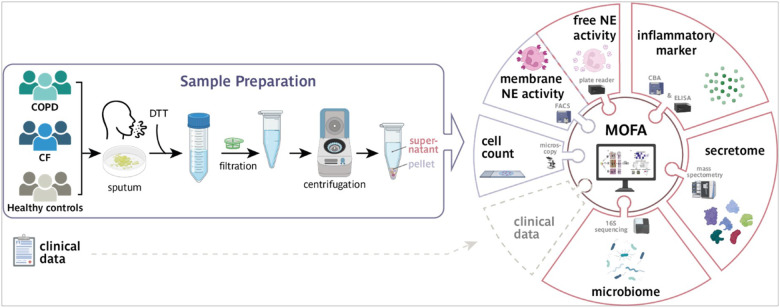
Overview of the SputOMICs workflow. The individual steps of sample processing and analysis as well as integration of clinical data and the multiomics data are schematically represented. Chronic obstructive pulmonary disease (COPD); dithiothreitol (DTT); neutrophil elastase (NE); flow cytometry (FACS); cytometric bead array (CBA); enzyme-linked immunosorbent assay (ELISA); Multi-omics factor analysis (MOFA).

Sputum samples were treated as described previously^24, 25^. In brief, spontaneously expectorated sputum from CF and COPD patients and induced sputum of healthy controls were divided into supernatant and cell pellet (Fig. 1). Samples were treated as previously described^26, 27^. Levels of endogenous anti-proteases (A1AT, A1AT/NE complex, SLPI, TIMP1, and LTB_4_ were quantified via ELISA (Abnova, R&D Systems, eBioscience). Inflammatory cytokines (IL-1α, IL-1β, IL-5, IL-6, IL-8, IL-10, TNF-α, TGF-β_1_ and IFN-γ) were quantified using cytokine bead arrays (BD Biosciences). Differential cell counts of May-Grünwald-Giemsa stained cell preparations determined percentage and total cell numbers.

### Free and surface-associated neutrophil elastase activity

Free and surface-associated neutrophil elastase (NE) activity was measured using Förster resonance energy transfer (FRET) based reporters NEmo-1 and NEmo-2^28^ (Sirius Fine Chemicals, Bremen, Germany) as previously described^24, 26, 27^, applying the recently introduced small molecule FRET flow procedure^24, 29^.

### Proteomic analysis by mass spectrometry

The protein content of thawed sputum supernatants was determined. Per sample, 5μg of protein was reduced, alkylated, digested, and cleaned up by an Auot-SP3 protocol and analyzed by an Ultimate 3000 HPLC-Orbitrap Exploris 480 mass spectrometer in data-independent mode. Spectronaut 15.6 was used with the UniProt Human-reviewed canonical reference proteome for data analysis. The mass spectrometric raw data and Spectronaut search files are available on proteomeXchange (PXD048388).

### Statistical analysis and multi-omics factor analysis

Statistical analyses were performed with R Statistical Software (v4.1.2; R Core Team 2021). Group-wise comparisons used a pairwise Wilcoxon rank sum test, adjusted for multiple comparisons with p-value < 0.05 considered significant. Kmeans clustering based on the Morisita-Horn dissimilarity index was defined as microbiome clusters. Proteomics data was analyzed with MSPypeline^30^, and the proteome, microbiome, and inflammatory marker dataset was integrated using the ‘MOFA2’ package (version 1.60)^31^.

## Results

### The SputOMICs workflow allows an integrative multilevel analysis of a study cohort.

To characterize changes in the sputum of CF and COPD patients and to uncover the complex interplay of multiple factors driving the diseases, we established a SputOMICs workflow (Fig. 1) combining diverse cellular and molecular profiles and integrates this multilevel analysis with clinical data. Sputum is a highly viscous fluid. Therefore, to make it accessible to the molecular studies in our workflow, it was homogenized using a 10% solution of Sputolysin (DTT), filtered, and centrifuged to generate a cellular pellet and a supernatant fraction (Fig. 1). The cellular pellet was used for cell typing, determining the number of inflammatory cells, and measuring the activity of membrane-associated Neutrophil Elastase (NE). Alterations in the microbiome, inflammatory factors, free NE activity, protease/antiprotease levels, and the proteome were analyzed in the supernatants. A Multi-Omics Factor Analysis (MOFA) was employed for the integrative data analysis to provide systems-wide insights into disease-relevant changes in the sputum. To evaluate our workflow, we performed a proof-of-concept study and examined sputum samples from a representative cohort comprising 38 CF patients, 18 COPD patients, and 10 healthy controls. The cohort captured diverse clinical and demographical features of both lung diseases as COPD patients were older (67 vs. 29 years), exhibited slightly higher BMI (22 vs. 21 kg/m^2^), and had a lower FEV_1_ percent predicted (34% vs 57%; GOLD stage III and IV) compared to CF patients (Table 1 and Supp. Figure 1A), enhancing the generalizability of our results. Spontaneous sputum from CF and COPD patients was used, while for the healthy controls, induced sputum was collected and separated from saliva.

In sum, our integrative SputOMICs workflow allows to examine both shared and unique alterations in the sputum of CF and COPD patients, and thus has the potential to establish promising sputum biomarkers for these diseases.

### Microbiome profiles are divergent in CF and COPD.

**Figure 2:**
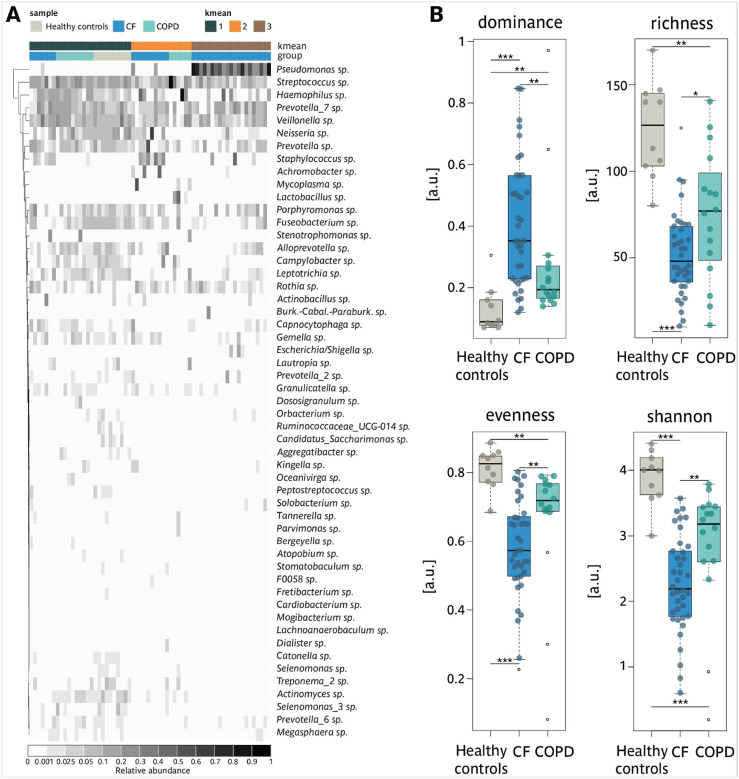
Comparison of microbiomes in sputum from patients with CF, patients with COPD and healthy controls. A Heatmap of the relative abundance of the top 53 ASVs, healthy controls; CF; COPD are indicated by color. The kmean cluster are indicated in teal, orange and brown. B Calculated biodiversity parameters: dominance, richness, evenness and Shannon index for each cluster. Healthy controls (•); CF (•); COPD (•), outliers are shown as empty circles (o).

The level of the respiratory microbiome is critical for shaping inflammation and structural changes in the respiratory tract, particularly in chronic respiratory diseases. While the analysis of sputum samples has much advanced our understanding of the role of the microbiome in CF, significantly less is known for COPD. To address this gap, we compared the sputum microbiomes of healthy controls, CF patients, and COPD patients using advanced 16S RNA sequencing (see Supp. Figures 2 and 3 for quality control of the sequencing). Our analysis identified three distinct microbiome clusters by k-means clustering (Fig. 2A and Supp. Figure 4). Microbiome cluster 1 encompassed all healthy controls, most COPD patients, and a subset of CF patients, while microbiome cluster 2 included additional COPD and CF patients. Microbiome cluster 3 was exclusively composed of CF samples. CF sputum microbiomes were dominated by well-known CF pathogens, with *Staphylococcus* (prevalent in microbiome clusters 2) and *Pseudomonas* (highly abundant in microbiome clusters 3). In contrast, COPD sputum microbiomes often resembled the structure of healthy controls (microbiome clusters 1) or were characterized by dominance of *Haemophilus, Streptococcus*, or *Lactobacillus* (microbiome clusters 2). Principal Coordinates Analysis (PCoA) revealed a distinct separation between the three microbiome groups (Supp. Figure 5). Biodiversity analysis underscored these differences. Healthy controls exhibited the most diverse microbiomes, characterized by low dominance, high richness and evenness, and the highest Shannon index (Fig. 2B). In contrast, CF sputum displayed the highest dominance and the lowest richness, evenness, and Shannon index (Fig. 2B). COPD microbiomes demonstrated intermediate diversity between the profiles of CF and healthy controls. Despite these differences, microbial copy numbers and biomass were comparable across groups (Supp. Figure 1D)

These findings underscore distinct microbiome differences between CF and COPD. CF is dominated by pathogenic bacteria like *Pseudomonas* and *Staphylococcus*, which drive inflammation and tissue damage, while COPD exhibits more diverse microbiomes linked to broader pathophysiological processes.

### Inflammatory profiles in CF and COPD sputum are distinct.

**Figure 3:**
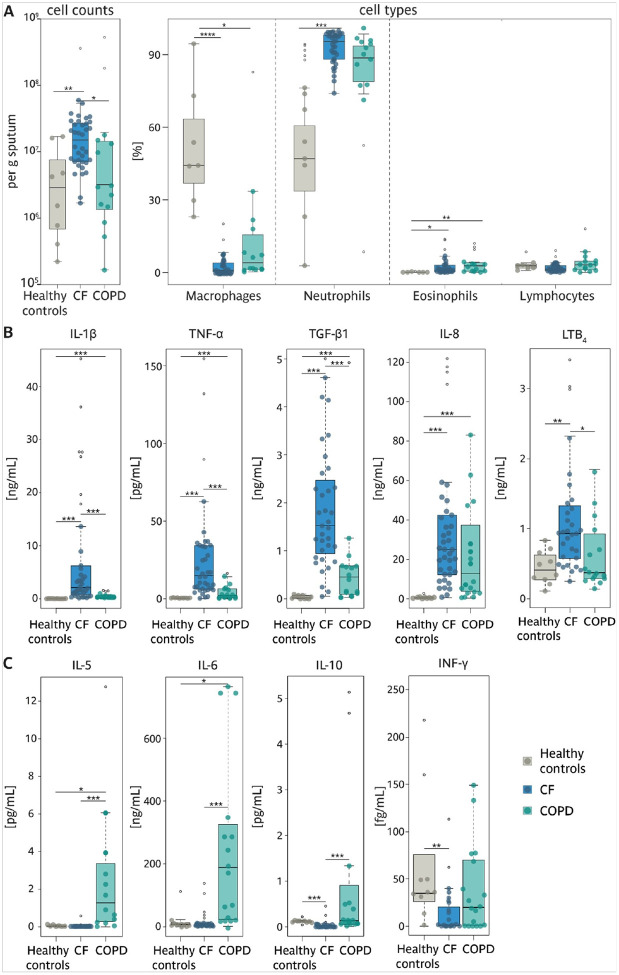
Total and differential inflammatory cell counts and inflammatory markers in sputum from patients with CF, patients with COPD, and healthy controls. Boxplot of A total inflammatory cells per gram sputum (healthy controls • n=8; CF • n=36; COPD • n=14, and differential cell count showing the percentages macrophages, neutrophils, eosinophils and lymphocytes (Healthy controls • n=7; CF • n=36; COPD • n=14. Boxplots of **B** IL-1β, TNF-α, TGF-β_1_, IL-8 and LTB_4_, C IL-5, IL-6, IL-10 and INF-γ are given. Healthy controls • n=8–10; CF • n=31–36; COPD • n=13–18, numbers per graph are given in the Supp. Tab. 1, outliers are shown as empty circles (o).

Based on the analysis of blood and sputum samples, chronic neutrophilic inflammation has been established as a common hallmark of both diseases^19, 20^, causing persistent respiratory symptoms and irreversible airflow limitation^32^. While in CF, inflammation is dominated by an overwhelming presence of neutrophils^33^, COPD exhibits a more heterogeneous pattern involving elevated eosinophils, macrophages, and lymphocytes^33, 34, 35, 36^. To better understand these differences, we analyzed the cellular fraction of our sputum samples (Fig. 3 and Supp. Figure 1B-C). We observed that total cell numbers were significantly higher in CF and moderately increased in COPD than in healthy individuals (Fig. 3A, left panel). Both diseases exhibited substantial neutrophilia, which was far more pronounced in CF. Interestingly, eosinophil levels were elevated in both diseases, while macrophage numbers were significantly reduced (Fig. 3A, right panel).

Despite these similarities in the cellular composition of the sputum, we found striking differences between the two diseases regarding the presence of inflammatory factors in the sputum supernatants (sample inclusion details are provided in Supplementary Table 1). CF samples had notably higher levels of key inflammatory markers such as IL-1β, TNF-α, TGFβ_1_, IL-8, and LTB_4_ (Fig. 3B), reflecting the intensity of neutrophilic inflammation in this condition. On the other hand, COPD samples showed elevated levels of IL-5, IL-6, and IL-10 (Fig. 3C).

These differences in inflammatory signatures point to a unique inflammatory environment in COPD.

### Protease-antiprotease balance is dysregulated in CF and COPD sputum.

**Figure 4:**
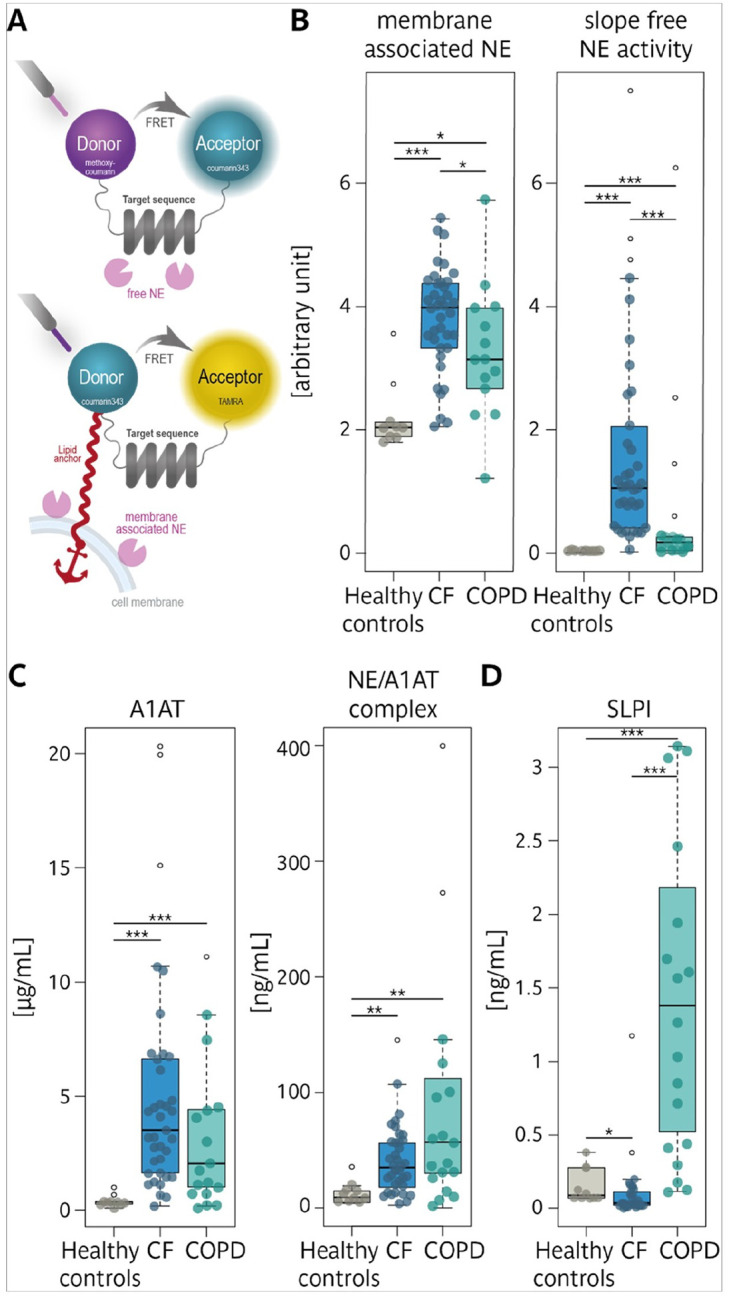
Comparison of membrane-associated and free NE activity, as well as levels of endogenous anti-proteases and complexes in sputum from patients with CF, patients with COPD and Healthy controls. **A** Principle of FRET reporters NEmo-1 for free NE activity and NEmo-2 for quantification of membrane-associated NE activity. Boxplots of **B** membrane-associated NE on the surface of sputum neutrophils, and slope of free NE activity in sputum supernatant **C** A1 AT and NE/A1 AT complex, and **D** SLPI. Healthy controls (•); CF (•); COPD (•), outliers are shown as empty circles (o).

For the integrity of the lung parenchyma, a balance between proteases and antiproteases is critical, and both diseases exhibit dysregulated protease activity, leading to tissue damage and chronic inflammation. However, the underlying mechanisms and severity of this imbalance could vary considerably between CF and COPD. The examination of the cellular sputum fraction showed that cell membrane-associated Neutrophil Elastase (NE) activity, a marker of neutrophil activation, was similarly elevated in both diseases, suggesting comparable levels of neutrophil-driven inflammation in CF and COPD (Fig. 4A–B, right panel; surface markers in Supp. Figure 1C). Yet surprisingly, free NE activity in sputum supernatants was dramatically higher in CF compared to COPD, indicating a severe breakdown of the anti-protease defense system in CF (Fig. 4A–B, left panel). Further analysis uncovered distinct protease-antiprotease patterns in the two diseases. In COPD, levels of the key inhibitor α−1-antitrypsin (A1AT) were increased, along with the formation of NE/A1AT complexes (Fig. 4C). In contrast, CF sputum showed reduced levels of Secretory Leukocyte Protease Inhibitor (SLPI) (Fig. 4D).

Thus, while CF is characterized by excessive protease activity and insufficient inhibition, COPD shows signs of an adaptive response, with increased inhibitor production to counterpart protease activity.

### Proteomic sputum profiling identifies distinct alterations in CF and high heterogeneity in COPD

**Figure 5:**
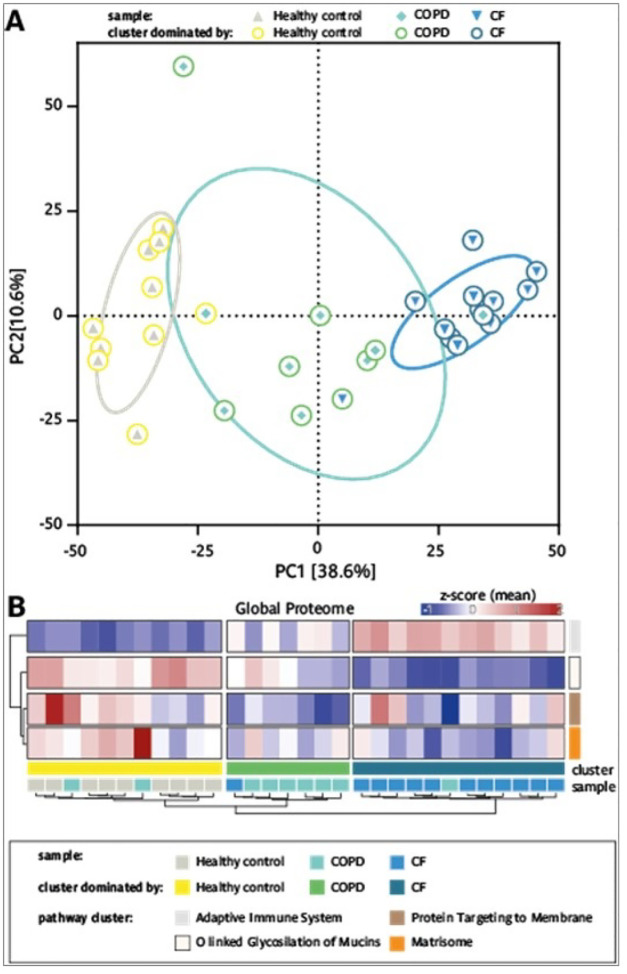
Comparison of sputum proteome of patients with CF, patients with COPD and Healthy controls. **A** Principal component analysis (PCA) of all detected proteins, circles indicate 50% of the 95% distribution interval. **B** Heatmaps of the global proteome analysis, top mean z-score of the four main protein clusters, and the most enriched pathway per cluster, as well as Healthy controls (•); CF patients (•); COPD patients (•).

**Figure 6:**
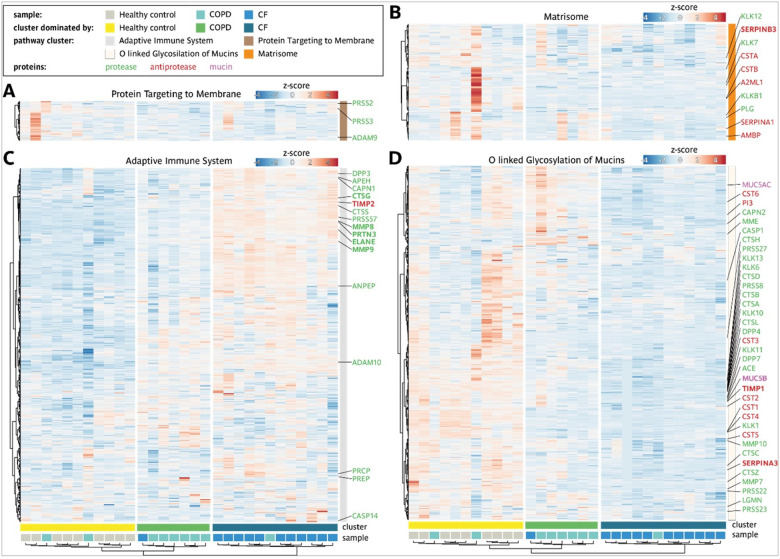
Detailed comparison of sputum proteome of patients with CF, patients with COPD and Healthy controls. Detailed heatmap of the main pathway clusters. All proteins which are proteases are indicated in green, antiproteases in red and mucin related proteins in purple. Sample cluster 1, C1 (•); sample cluster 2, C2 (•); sample cluster 3, C3 (•). Healthy controls (•); COPD (•) and CF (•).

**Figure 7:**
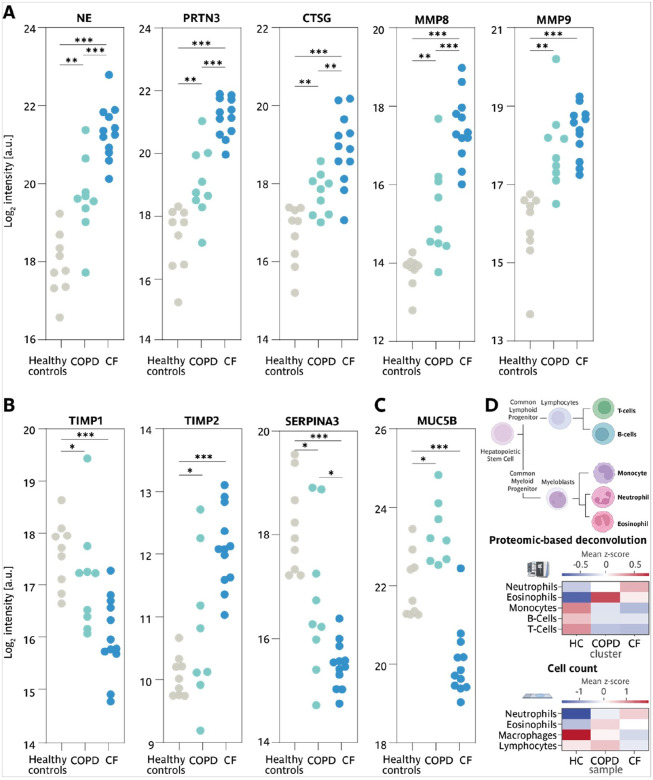
Comparison of proteases, antiproteases and mucins as well as proteomic cell deconvolution from the sputum of patients with CF, patients with COPD and Healthy controls. **A** Confetti plots displaying the log2 intensities of individual proteins per group. Neutrophil elastase (NE), Myeloblastin (PRTN3), Cathepsin G (CTSG), Matrix metalloproteinases (MMP) 8 & 9. **B** Metalloproteinase inhibitor (TIMP) 1 & 2, α1antichymotrypsin (SERPINA3), **C** Mucin-5B (MUC5B). **D** Tree of the hematopoietic Stem Cell derived immune cell types, analyzed cell types are labeled in bold, average of the mean z-score of specific log2 protein intensity (upper panel) and mean z-score of the different immune cell counts (lower panel). Healthy controls (•); CF patients (•); COPD patients (•).

To provide a systems-wide view of disease-specific molecular alterations in CF and COPD and elucidate the underlying mechanisms contributing to these chronic respiratory diseases, we employed a mass spectrometry-based proteomics approach utilizing data-independent acquisition (DIA). A total of 1,495 proteins were identified and quantified across sputum supernatants from healthy individuals, CF patients, and COPD patients (Supp. Figure 6B), with an average of 1,381 proteins detected per sample. Downstream data processing and bioinformatics analysis was performed using the MSPypeline^30^ to ensure robust and reliable statistical interpretation. Principal component analysis (PCA) of the proteomics data from healthy, CF, and COPD samples revealed significant differences between the proteomes of healthy individuals and CF patients. Strikingly, alterations in the sputum proteome of COPD patients were more heterogenous; as for some patients, it overlapped with healthy profiles, for others with CF, and for most it formed an intermediate group (Fig. 5A). These patterns highlighted that diverse molecular alterations may contribute to COPD. To characterize pathways differentially regulated in both respiratory diseases, protein expression profiles from healthy controls, CF and COPD patients were analyzed using String Pathway Analysis. The differentially expressed proteins clustered into four distinct groups, each associated with a specific biological pathway: the adaptive immune system, O-linked glycosylation of mucins, protein targeting to membranes, and the matrisome (a collection of extracellular matrix proteins) (Supp. Figure 6A ). To quantify per sample the contribution of pathway alterations, mean zscores were calculated, summarizing changes in the pathway-specific proteins (Fig. 5B). The results showed that regarding the top regulated pathways the proteomics profiles of the samples formed three clusters: A cluster dominated by healthy controls, a cluster dominated by COPD and a cluster dominated by CF. While in the cluster dominated by CF an upregulation of adaptive immune system and a downregulatiorn of mucin glycosylation prevailed, in the cluster dominated by COPD exhibited a more heterogeneous response, characterized by a reduction in Signal Recognition Particle (SRP)-dependent protein targeting to the membrane and a trend towards a decrease in the matrisome (Fig. 5B). The comparision of the relative changes in proteins contributing to the top regulated pathways showed that for the three clusters, dominated by healthy controls, CF and COPD patients, characteristic changes in the balance of proteases and anti-proteases were observed. Specifically, for the pathway ‘protein targeting to membrane’ a trend towards changes in the proteases Trypsin-2 (PRSS2), Trypsin-3 (PRSS3) and Disintegrin and metalloproteinase domain-containing protein 9 (ADAM9) was identified, while for the ‘matrisome’ pathway changes in the balance of certain proteases and antiproteases prevailed (Fig. 6A,B). In particular for the CF dominated cluster, a strong upregulation of multiple proteases contributing to the pathway ‘adaptive immune system’ pathway (Fig. 6C) and a down regulation of many proteases and antiproteases associated with the pathway ‘O linked glycosylation of mucin’ pathway and of several mucins (Fig. 6D) was observed. To identify disease specific differences in the expression levels of key proteins, we compared the individual protein intensities. Our analysis showed that proteases such as neutrophil elastase (NE), proteinase 3 (PRTN3), and cathepsin G (CTSG), and matrix metalloproteinases (MMP) 8 and 9, were significantly more abundant in CF sputum and only increased to a lesser extent in COPD. Inhibitory proteins like α1antichymotrypsin (SERPINA3) and TIMP1 were low in CF and intermediate in COPD, while TIMP2 was significantly elevated in CF while varying in COPD (Fig. 7A–B). Mucin-5B (MUC5B), the key mucin, was reduced in CF, but increased in COPD, thus further highlighting differences in mucus composition (Fig. 7C).

Thus, the identified disease specific changes in the sputum proteome indicate a key role for mechanisms leading to impaired mucus properties in CF and underscore the importance of tissue remodeling in COPD.

### Proteome-based cellular deconvolution points to a major contribution of eosinophils in COPD

Despite the common upregulation of neutrophils that we detected by cell counting in both chronic respiratory diseases, we observed major differences in the pattern of inflammatory factors present in the sputum of CF and COPD patients. To resolve this discrepancy, we tested whether we could utilize our detailed proteome-wide characterization of the sputum samples to deconvolute the cellular composition and focus in light of the major impact of inflammatory processes on the presence of immune cells. In analogy to the deconvolution algorithms and gold-standard datasets that have been developed for RNA sequencing, we utilized curated marker lists from the Human Protein Atlas, ensuring that all selected markers are supported by proteomic evidence. Applying this approach to our sputum proteomes, we estimated the relative contributions of immune cell populations. In line with the mean cell counts we determined, the proteome-based deconvolution provided evidence for an upregulation of neutrophils and esinophils in both chronic respiratory diseases and a downregulation of macrophages and B and T-cells.

Interestingly, these high-resolution examinations showed that while in CF the upregulation of neutrophils dominated, in COPD an increase in eosinophils prevailed.

These findings demonstrate that, based on the global proteome information, the average cellular composition can be deduced and point to a distinct immune landscapes in CF and COPD: While both chronic respiratory diseases are characterized by neutrophilia, COPD presents a more complex inflammatory profile with elevated eosinophils resulting in a unique cytokine signature, and revealing that fundamentally different mechanisms drive these diseases.

### Integrative multi-omics factor analysis ranks contributions to disease phenotypes.

**Figure 8:**
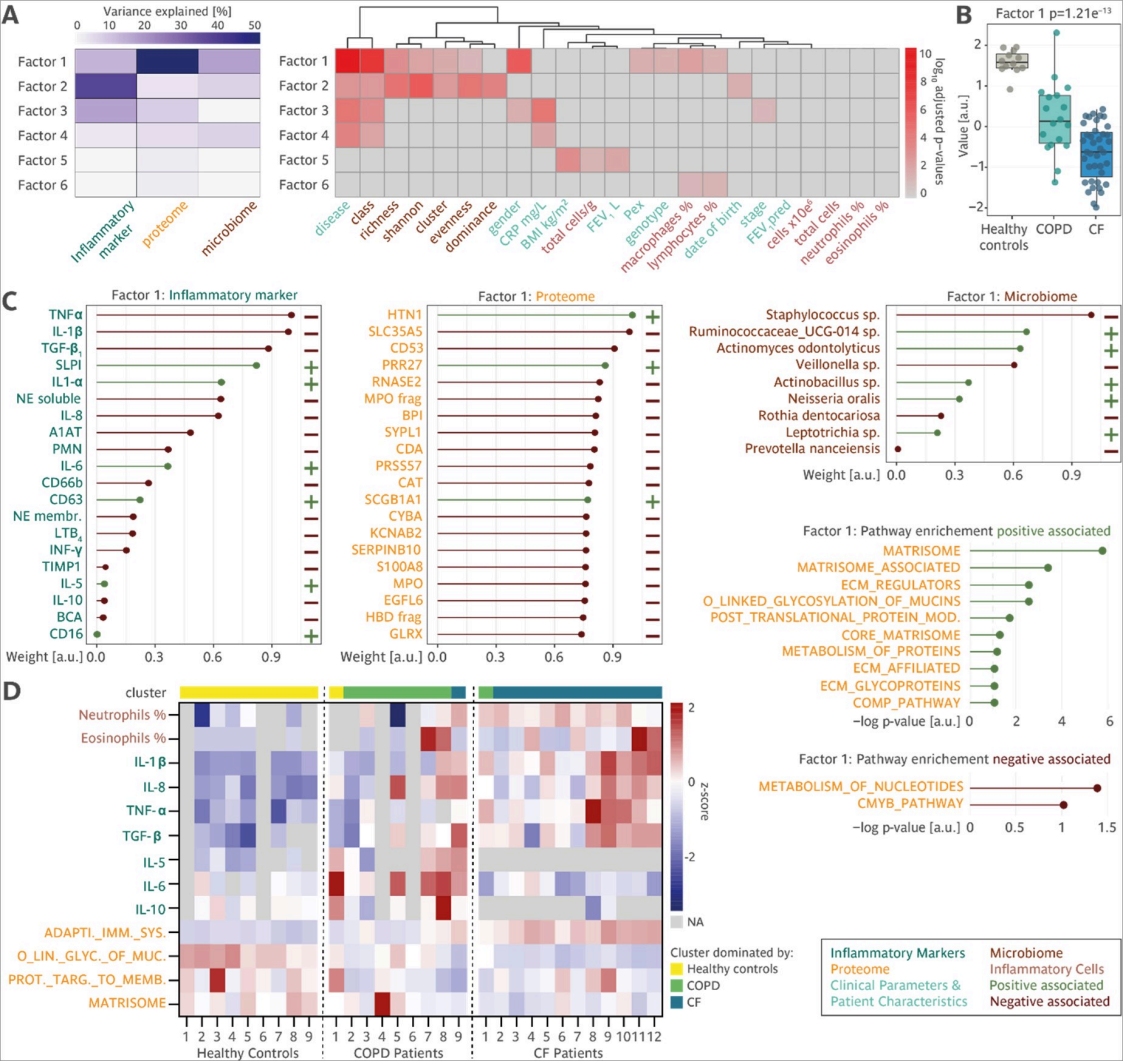
Multi-omics factor analysis of inflammatory markers, microbiome and proteomics in sputum from patients with CF, patients with COPD and Healthy controls. **A** Explained variance for each omic dataset by each MOFA factor, heatmap showing the association p-values between MOFA factors and sample metadata. For continuous metadata features (e.g. BMI, CRP, total cells etc.), p-values were calculated using Pearson’s correlation tests. For categorical metadata features (e.g. gender, stage and so on), ANOVA test was used. **B** Boxplot showing the distribution of factor 1 values in the Healthy controls, CF patients, and COPD patients. P-value was calculated by ANOVA test. **C** Loadings (weights) of top features from the inflammatory marker (left), proteome (middle) and microbiome (right) of factor 1 from the MOFA. **D** Pathways enriched for the proteins (from the proteomic view) positively (left) or negative (right) correlated with factor 1. Enrichment p-values were calculated using gene set enrichment analysis (GSEA) against the human cancer hallmark gene sets from the molecular signatures database (MSigDB). **E** Heatmaps depicting the percentages of neutrophils and eosinophils, inflammatory markers (IL-1β, IL-8, TNF-α, TGF-β_1_, IL-5, IL-6, IL-10), and the top regulated pathways from the global proteome analysis (adaptive immune system, O-linked glycosylation of mucins, SRP-dependent cotranslational protein targeting to membrane, and the matrisome). The order of the patients per group is based on the PCA1 score from the global proteome analysis, the sample cluster of the individual samples is indicated on top. Heatmap cells with missing values are indicated depicted in light grey.

To gain insights into the complex interplay between microbial, molecular, and inflammatory processes in CF and COPD, an integration and comparison of the multi-level sputum data from healthy controls, CF patients, and COPD patients is required. Therefore, we applied MOFA, an unsupervised computational framework designed to integrate heterogeneous omics datasets^31^. MOFA enables the identification of key sources of variation across different molecular layers and ranks their relative impact, providing a systematic approach to uncovering disease-specific molecular signature and their contributions. Through integrating microbiome (13 species of 64 participants), proteomic (987 proteins of 30 participants), and inflammatory marker data (20 factors of 65 participants) (Supp. Figure 9A), MOFA identified six factors that explain at least 1% variance in any omic data (Fig. 8A, left panel). Factor 1 was primarily driven by proteomic changes, but also reflected alterations in inflammatory markers and the microbiome. This factor showed a strong association with microbial classes and patient gender and was most effective in distinguishing healthy controls, CF patients, and COPD patients, making it the most predictive factor for the respective disease (Fig. 8B). Factor 2 was also informative for distinguishing CF and COPD as it was predominantly influenced by inflammatory markers and correlated with microbiome diversity metrics, including Shannon index, richness, evenness, and dominance (Fig. 8A, right panel). The remaining factors played a less important role in differentiating CF from COPD (Supp. Figure 9B-F). MOFA not only enables the identification of key diseases-specific patterns, but also allows for a detailed evaluation of the contribution of individual omic features and pathways regarding their direction and weight. Features with positive weigths on a certain factor are those whose abundance are elevated in samples where this factor value is high while negative weights indicate a lower abundance in samples where the factor value is high. The absolute value of the weight of a individual feature indicates their importance. Factor 1 describes a gradient from healthy controls, COPD and CF with the factor values ranging from high to low. Deconstructing factor 1 provided evidence for distinct inflammatory and proteomic signatures in CF and COPD (Fig. 8C). Among inflammatory markers, TNF-α, IL1β, and TGF-β_1_, were the most significant negative contributors, reflecting their higher abundance in CF patients compared to COPD patients and healthy controls. In contrast, SLPI, a key anti-inflammatory molecule, was the strongest positive contributor, indicating higher levels in COPD and healthy controls than CF patients. Within the proteome, histatin-1 (HTN1), proline-rich protein 27 (PRR27), and uteroglobin (SCGB1A1) were positive contributors, showing increased abundance in COPD and healthy controls. Conversely, negative contributors included solute carrier family 35 member A5 (SLC35A5), leukocyte surface antigen (CD53), and ribonuclease (RNASE2), which were more abundant in CF patients. In the microbiome, *Staphylococcus sp*. and *Veillonella sp*. emerged as the most negatively associated features, indicating its predominance in CF samples. Whereas *Ruminococcaceae_UCG-014 sp*. and *Actinomyces odontolyticus* had the strongest positive association, indicating a higher abundance in healthy control samples. Pathway enrichment analysis further highlighted the functional implications of factor 1. Positively associated proteins were enriched in the matrisome, extracellular matrix (ECM), and mucin-related pathways, underscoring their role in maintaining structural integrity and mucus properties which is impaired in patients compared to healthy controls. In contrast, negative associations were observed with nucleotide metabolism and the c-MYC pathway, suggesting distinct metabolic and cellular activity profiles in disease states (Fig. 8C). Interestingly, sorting of COPD patients based on their proteomic profiles with regards to similarities to healthy controls or CF patients revealed a gradient of increasing similarities to CF with respect to changes in cell counts, inflammatory factors and pathways, highlighting the heterogeneity of COPD patients and the possibility for subgrouping (Fig. 8D).

## Discussion

There is a growing clinical need for a reliable, and non-invasive method to assess lower airway health, particularly for millions of COPD patients, many of whom are in poor general health^3^. Traditional diagnostics, such as bronchoscopy or lung biopsies, are invasive, resource-intensive, and poorly tolerated, especially in severely ill or elderly patients. Therefore, developing accessible, patient-friendly diagnostic tools is crucial to improve disease monitoring^14^, early intervention, and personalized treatment strategies. To address this, we introduce the SputOMICs workflow, a robust multiomics pipeline that integrates clinical data, to uncover disease-specific microbiome, inflammatory, and proteomic signatures in the sputum. By systematically analysing molecular and cellular alterations, we revealed distinct mechanisms driving CF and COPD pathophysiology, with implications for patient stratification and therapeutic targeting.

Sputum is an easily accessible clinical specimen, offering airway-specific insights by capturing inflammatory cells, microbes, and markers embedded in highly viscous mucus; providing greater diagnostic value than blood-based analyses. To ensure efficient sputum lysis and cellular integrity, we developed a standardized preparation protocol within the SputOMICs workflow, which separates sputum into two distinct fractions: the supernatant and the cell pellet, each offering complementary disease insights. The cell pellet retains viable neutrophils, enabling NE activity assessment and differential cell count via H&E staining, while the supernatant allows the analysis of microbiome composition, inflammatory markers, and the proteome, providing a comprehensive molecular and cellular profile of the airway environment. Notably, bulk proteomics-based cellular deconvolution aligned strongly with the H&E-based cell counts, despite being derived from different fractions. This agreement underscores the robustness of our workflow in accurately capturing the sputum cellular landscape and highlights proteomic deconvolution as a powerful alternative to traditional cell quantification methods.

Our microbiome analysis uncovered striking differences between CF and COPD. The CF sputum was dominated by persistent pathogens such as *Pseudomonas aeruginosa* and *Staphylococcus*, which are strongly associated with chronic infection and progressive lung damage^37, 38^. In contrast, COPD microbiomes were more diverse, often resembling healthy profiles or dominated by bacteria such as *Haemophilus, Streptococcus*, or *Lactobacillus*. This suggests that microbiome diversity in COPD could serve as a potential biomarker for disease severity and progression and that preserving or enhancing microbiome diversity could be a promising therapeutic avenue. In CF, the persistent pathogen-dominated microbiome underscores the potential for microbiome-targeted therapies, including probiotics or bacteriophage treatments, to reduce pathogenic dominance^39^.

In accordance with the chronic neutrophilic inflammation characteristic of CF and COPD, sputum from both disease harbors elevated neutrophil and eosinophil levels. However, proteome-based cellular deconvolution revealed a disease-specific shift: CF sputum presented a stronger neutrophilic increase, whereas COPD exhibited a predominance of eosinephils, potentially explaining their distinct inflammatory profiles. CF sputum displayed a more aggressive inflammatory phenotype, marked by pronounced neutrophilia, elevated IL-1β, TNFα, TGF-β_1_, and reduced IFN-γ levels. In contrast, COPD sputum featured elevated IL-5, IL-6, and IL-10, suggesting a more heterogeneous inflammatory environment. These findings align with previous studies that identify CF as predominantly Th1-driven, while COPD inflammation reflects a mix of Th2 and regulatory responses^40, 41^. The distinct inflammatory pathways present opportunities for tailored therapeutic interventions, such as cytokine modulators in CF or targeting IL-6 or IL-10 in COPD to mitigate excessive inflammation.

Both CF and COPD showed dysregulated protease-antiprotease activity, a hallmark of chronic respiratory diseases^2, 3, 29, 36^. However, their distinct patterns highlight diverging pathophysiological trajectories. CF sputum showed markedly elevated NE activity and reduced SLPI levels, exacerbating proteolytic imbalance and airway damage. Conversely, while NE activity was also elevated in COPD, compensatory mechanisms, such as increased A1AT and NE/A1AT complexes, mitigates its effects. These findings suggest that protease inhibitors could be particularly beneficial in CF patients, whereas restoring the protease-antiprotease in COPD may complement anti-inflammatory therapies, reducing airway injury.

The SputOMICs workflow encompasses high-depth proteomic profiling of sputum through an optimized lysis protocol and a semi-automated SP3-based pipeline^42^ with data-independent acquisition (DIA) mass spectrometry, allowing for the robust identification of 1,500 proteinsa significant advancement in non-invasive respiratory disease diagnostics. This high-throughput approach requires minimal sample material, reduces manual bias, and ensures scalability for clinical implementation. Unlike lung tissue biopsies, which are invasive and impractical for routine monitoring, sputum provides a readily accessible, non-invasive alternative, capturing inflammatory mediators, immune cells, and microbial signatures. Previous sputum proteomics studies have yielded valuable insights, yet with lower protein coverage; Yan *et al*. reported 280 proteins in COPD sputum, highlighting microbiome-host interactions^21^, while Maher *et al*. demonstrated CFTR modulator therapy-induced proteomic shifts, with 80 proteins increasing and 30 decreasing post-therapy^18^. Volpato *et al*. linked sputum rheology to eosinophilic inflammation, underscoring its potential as a disease biomarker^35^. Although tissue-based proteomics has provided critical insights—Ohlmeier *et al*. identified 82 altered proteins in lung tissue^17^, while Titz *et al*.^43^ and Schiller *et al*.^44^ characterized smoking-induced proteomic changes and lung injury repair, our study bridges the gap between tissue and airway proteomics. We identified CF-specific enrichment of adaptive immunity and mucin glycosylation proteins, while COPD sputum exhibited downregulation of SRPdependent protein targeting and extracellular matrix components, reflecting impaired tissue remodeling. Importantly, our deep proteomic coverage uncovered distinct COPD patient subgroups, with molecular profiles resembling either healthy controls or CF patients, reinforcing COPD heterogeneity and underscoring the potential of proteomics to refine disease classification and patient stratification. By integrating the proteomics workflow automation, high-throughput data acquisition, and broad molecular coverage, the SputOMICs current pipeline establishes a robust, scalable, and clinically relevant platform for for precision medicine applications in CF, COPD, and related diseases.

Cellular composition of sputum is highly informative for therapeutic decision-making. Using a cellular deconvolution approach previously developed for genomic and transcriptomic data^45^, we demonstrated that bulk proteome data can accurately determine sputum cell types. Unlike traditional H&E-based cell counts, which require manual counting of 400 cells, mass spectrometry-based deconvolution provides a high-throughput, less biased alternative, capable of identifying multiple sub-cellular populations that are otherwise difficult to distinguish. Moreover, rare or low-abundant cell types, often underrepresented in manual counts, are better captured, allowing a more comprehensive and quantitative assessment of the cellular landscape. This effect was particularly evident for eosinophils, which appeared underrepresented in traditional cell counts but show the strongest differences between CF and COPD in proteomic deconvolution analysis. The elevated IL-5 levels detected in COPD, a cytokine essential for eosinophil maturation and survival^13, 33^, support the role of eosinophils in COPD pathophysiology. Notably, IL-5 has been previously reported to be elevated in bronchoalveolar lavage fluid of patients with high eosinophil counts in the blood^33^, further reinforcing the validity of our findings.

Integrating multi-omics data using MOFA provided key insights into the molecular and microbial drivers of disease phenotypes. Factor 1, primarily influenced by the sputum proteome, was strongly linked to microbial composition and inflammatory diversity, and most effectively distinguishing healthy controls, CF, and COPD patients. Pathway enrichment analysis revealed CF-specific upregulation of mucin-related pathways, whereas COPD showed alterations in matrisome composition and reductions in nucleotide metabolism as well as cMYC pathways. These findings underscore the potential of integrated multi-omics approaches to uncover actionable biomarkers and improve patient stratification.

This study serves as a proof-of-concept for the potential of an integrative multi-omics approach, yet limitations exist. The relatively small sample size, single-center design, and age differences between CF and COPD cohorts may impact generalizability of our findings. Additionally, the cross-sectional design prevents causal inferences, emphasizing the need for longitudinal studies to validate findings and explore their clinical implications. Future research should focus on expanding cohorts, integrating longitudinal data, and validating the identified biomarkers to advance precision medicine for CF and COPD.

This study highlights the value of an integrative multi-omics approach in uncovering disease-specific microbiome, inflammatory and proteomic profiles in CF and COPD. Our findings provide a foundation for more precise disease classification and personalized therapeutic strategies by identifying key markers and pathways unique to each disease. Future research should prioritize validating these sputum markers in larger, longitudinal cohorts and developing personalized interventions that address the specific pathophysiological mechanisms identified here. Such efforts could revolutionize the management and treatment of chronic respiratory disease and improve the outcomes for patients with CF and COPD.

## Supplementary Files

This is a list of supplementary files associated with this preprint. Click to download.

- submissionSupplementNatCom.docx

## Figures and Tables

**Table 1:** Demographics and clinical characteristics of study population.

		CF	COPD	Healthy controls
**Subjects**	n	38	18	10
**Age (years)**	Median (range)	29.33 (20.80–73.83)	66.57 (50.70–78.20)	30.68 (27.32–49.04)
**Sex**	n, females/males	11/27	10/8	5/5
**BMI (kg/m^2^)**	Median (range)	20.96 (15.79–30-96)	21.96 (18.59–44.46)	
**FEV**_**1**_ **% predicted** *	Median (range)	57.19 (16.93–92.88)	34.10 (18.30–47.70)	
**CFTR genotype**				
F508del/F508del	n (%)	14 (36.84)	-	-
F508del/other	n (%)	20 (52.63)	-	-
Other/other	n (%)	4 (10.53)	-	-
**Pancreatic insufficiency**	n (%)	35 (92.11)	-	-
**GOLD stage (3/4)**	n (%)	-	12(66.66)/6(33.33)	-
**RV % predicted**	Median (range)	-	208.50 (170.3–288.1)	-
**Diff % predicted**	Median (range)	-	34.15 (21.50–74.20)	-
**6 minute walking test (m)**	Median (range)	-	328.50 (152.00–432.0)	-

* FEV_1_% predicted only available from 32 CF patients

## Take home message

Integrative multi-omics sputum analyses identify distinct patterns of airway inflammation, microbiome, and proteome alterations in CF and COPD. These disease-specific sputum markers could facilitate the monitoring of treatment responses in CF and improve diagnosis and therapeutic decisions in COPD.
